# CMR detects a reduction in infarct size and myocardial edema when primary PCI is augmented by Remote Ischemic Conditioning. A randomized trial

**DOI:** 10.1186/1532-429X-16-S1-P208

**Published:** 2014-01-16

**Authors:** Steven K White, Georg M Frohlich, Daniel Sado, Viviana Maestrini, Marianna Fontana, Thomas A Treibel, Shana Tehrani, Heerajnarain Bulluck, Andrew S Flett, Pascal Meier, James Moon, Derek Yellon, Derek J Hausenloy

**Affiliations:** 1 The Hatter Cardiovascular Institute, London, UK; 2 The Heart Hospital, London, UK; 3 University Hospital Southampton NHS Foundation Trust, Southampton, UK

## Background

CMR is the imaging modality of choice to quantify myocardial injury in studies of cardioprotection. Remote ischemic conditioning (RIC), using transient limb ischemia and reperfusion, is a novel therapeutic intervention, which can protect the heart against acute ischemia-reperfusion injury (IRI). Whether RIC can reduce myocardial infarct (MI) size, and improve myocardial salvage in ST-segment elevation myocardial infarction (STEMI) patients treated by primary percutaneous coronary intervention (PPCI), is unknown, and was investigated in this randomized control clinical trial using CMR.

## Methods

323 consecutive patients with suspected STEMI were screened and randomized to receive either RIC (four-5 minute cycles of upper-arm cuff inflation/deflation) or control (un-inflated cuff) prior to PPCI. 197 met study inclusion criteria of confirmed STEMI with TIMI 0 flow. The primary study endpoint was MI size, measured by late gadolinium enhancement (LGE) on day 3-6. Myocardial edema was quantified for the first time in a clinical trial by T2 mapping, the extent of edema representing the area-at-risk (AAR). T2 values were assessed in remote myocardium and the area-at-risk, providing an additional surrogate marker for the amount of edema present. The Otsu thresholding technique was pre-validated as the most reproducible technique for quantification of both MI and T2 edema compared to 8 other techniques (manual, fwhm, huang, 2-6 sd).

## Results

RIC reduced MI size by 27% (18.0 ± 10% versus 24.5 ± 12.0%; P = 0.009). However, RIC also reduced the extent of myocardial edema measured by T2-mapping CMR (28.5 ± 9.0% versus 35.1 ± 10.0%; P = 0.003), and lowered mean T2 values (68.7 ± 5.8 ms versus 73.1 ± 6.1 ms; P = 0.001), precluding the use of CMR edema imaging to correctly estimate the area-at-risk (AAR). Using CMR-independent coronary angiography jeopardy scores to estimate the AAR, RIC was found to significantly improve the myocardial salvage index (MSI) when compared with control (0.42 ± 0.29 versus 0.28 ± 0.29; P = 0.03).

## Conclusions

In STEMI patients treated by PPCI, remote ischemic conditioning, initiated prior to PPCI, reduced myocardial infarct size and increased myocardial salvage. Unexpectedly, RIC also reduced the extent of myocardial edema and lowered T2 values. This supports the cardioprotective efficacy of RIC, but importantly, adds to current controversy questioning the use of T2-CMR to estimate the AAR.

## Funding

Dr White is supported by a Clinical Research Training Fellowship from the British Heart Foundation (grant FS/10/72/28568).This work was also supported by the British Heart Foundation (grant numbers: RG/03/007, FS/10/039/28270), the RoseTree Trust, and the National Institute for Health Research University College London Hospitals Biomedical Research Centre. Dr Frohlich is supported by a research grant from the Swiss National Foundation.

**Figure 1:**
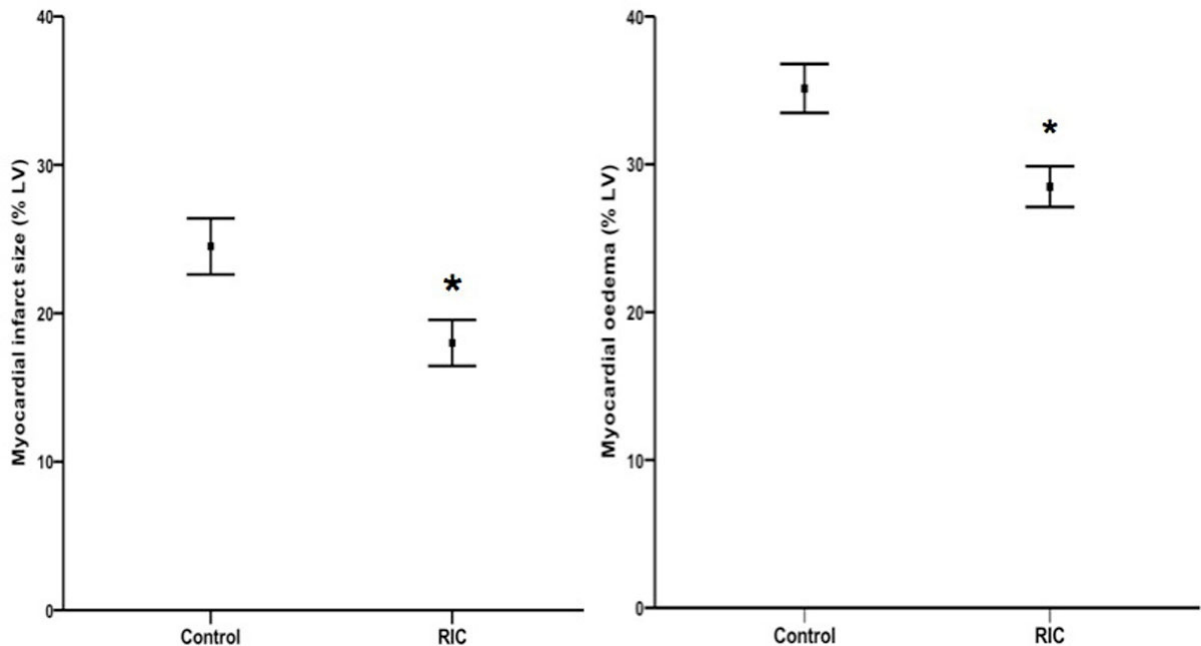
**a. RIC reduced infarct size**. Patients randomized to RIC sustained a significantly smaller myocardial infarct size (% LV). Values are mean ± SEM. *P = 0.009. Figure 1b. RIC reduced extent of edema. Unexpectedly, in patients randomized to RIC the extent of myocardial edema delineated by quantitative T2-mapping (% LV) was significantly reduced. Values are mean ± SEM. *P = 0.003.

**Figure 2:**
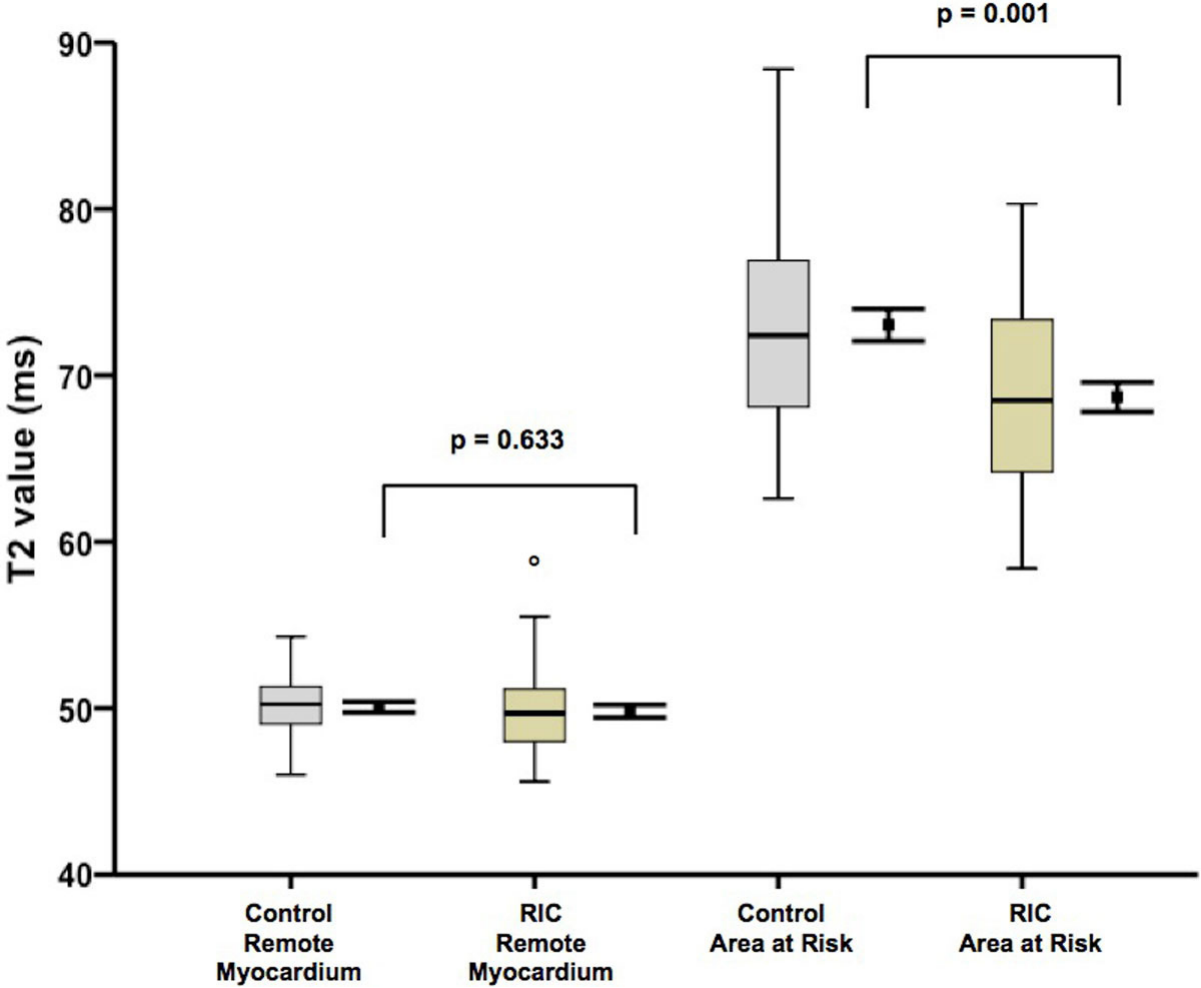
**RIC reduced T2 values in the AAR**. In patients randomized to RIC mean T2 values in the area-at-risk were significantly reduced when compared to control patients (p = 0.001). However, there was no significant difference in mean T2 values in the remote, non-infarcted myocardium (p = 0.633). Values are mean ± SEM (Error bars) and median + IQR (Box plot). ° one outlying patient.

